# The Effectiveness of Contact Tracing in Emerging Epidemics

**DOI:** 10.1371/journal.pone.0000012

**Published:** 2006-12-20

**Authors:** Don Klinkenberg, Christophe Fraser, Hans Heesterbeek

**Affiliations:** 1 Theoretical Epidemiology, Department of Farm Animal Health, Faculty of Veterinary Medicine, Utrecht University Utrecht, Netherlands; 2 Department of Infectious Disease Epidemiology, Imperial College London London, United Kingdom; University of California, Berkeley, United States of America

## Abstract

**Background:**

Contact tracing plays an important role in the control of emerging infectious diseases, but little is known yet about its effectiveness. Here we deduce from a generic mathematical model how effectiveness of tracing relates to various aspects of time, such as the course of individual infectivity, the (variability in) time between infection and symptom-based detection, and delays in the tracing process. In addition, the possibility of iteratively tracing of yet asymptomatic infecteds is considered. With these insights we explain why contact tracing was and will be effective for control of smallpox and SARS, only partially effective for foot-and-mouth disease, and likely not effective for influenza.

**Methods and Findings:**

We investigate contact tracing in a model of an emerging epidemic that is flexible enough to use for most infections. We consider isolation of symptomatic infecteds as the basic scenario, and express effectiveness as the proportion of contacts that need to be traced for a reproduction ratio smaller than 1. We obtain general results for special cases, which are interpreted with respect to the likely success of tracing for influenza, smallpox, SARS, and foot-and-mouth disease epidemics.

**Conclusions:**

We conclude that (1) there is no general predictive formula for the proportion to be traced as there is for the proportion to be vaccinated; (2) variability in time to detection is favourable for effective tracing; (3) tracing effectiveness need not be sensitive to the duration of the latent period and tracing delays; (4) iterative tracing primarily improves effectiveness when single-step tracing is on the brink of being effective.

## Introduction

Control of epidemics of (emerging) infectious diseases, such as SARS, pandemic influenza, or foot-and-mouth disease, always faces the difficulty that some infectives are not yet observed. By concentrating control measures only on observed cases (treatment, isolation, culling), resources are used efficiently but control is often not effective enough. On the other hand, by directing control to the whole population (mass vaccination, prophylactic treatment, preventive culling), epidemics are most likely contained, but at high cost.

Contact tracing of symptomatic infecteds works on an intermediate level: treatment or quarantine of contactees (by *contact* we mean the possible transmission event, by *contactee* the individual that is contacted) may be effective because unidentified infecteds are most likely to be found among contactees, and efficient because the resources can be directed towards individuals at risk only. Tracing and quarantine has been successfully applied for smallpox control, where the term ‘Leicester method’ refers to exactly this strategy, with the establishment of specific smallpox hospitals [1]. It was also successful during the more recent SARS epidemic [2], but not during the British foot-and-mouth epidemic [3]. Here we wish to investigate how these differences can be accounted for by the tracing process, thereby distinguishing tracing and quarantine as having separate effects which can be explained as follows.

Symptoms and detection divide the reproduction ratio *R* (the average number of secondary infecteds per primary infected in a susceptible population) into a part before detection and isolation and a part after [4]:

*R = R_0_^pre^+c (R_0_−R_0_^pre^)*,

where *c* is the reduction factor due to isolation, *R*_0_ is the *basic* reproduction ratio, when no efforts are made to isolate, and *R*_0_ *^pre^* is that part of *R*_0_ occurring prior to detection. Contact tracing will lead to earlier prevention of transmission due to quarantine of traced infecteds, thereby reducing the uncontrolled transmission *R*_0_ *^pre^* to *R^pre^*, and the reproduction ratio *R* to *R_q_*:

*R_q_* = *R^pre^*+*c* (*R*_0_−*R^pre^*).

The effect of decreasing *R*_0_ *^pre^* to *R^pre^* is distinct from the effect of isolation and quarantine which reduce *c*. For tracing to be effective, *R_q_* should be smaller than 1, so *R^pre^* should be smaller than (1−*c*)/(1−*cR*_0_), a threshold value determined by *c* and *R*_0_ [4]–[6]. For comparison of contact tracing in different situations, we will choose *c* = 0 (as in [6]–[10]), because it is easy and as arbitrary as any other value. The value *c* = 0 is valid for foot-and-mouth disease where traced herds are culled.

Several tracing studies have been published, some focussing on specific infections [10], [11] and some with the objective to obtain more general insight, e.g. in a general Markov-type SIR model [7], in a model incorporating symptom development [4], or in simulation models with specific network contact structures [8], [9]. Different assumptions were made with regard to the possibility of only tracing contacts of symptomatic infecteds (single-step tracing [4], [10], [11]), or of iteratively tracing the contacts of traced infecteds (iterative tracing [6]–[9]). However, no general insight has yet been obtained in reducing *R^pre^* in relation to time-related characteristics of the infection and the tracing process, such as the latent and infectious periods, the time of symptom-based detection, and delays in the tracing process.

In this paper we consider *detection* and *isolation* as an autonomous process most likely governed by detection via clinical symptoms. This allows us to concentrate on *contact tracing* and *quarantine*, which are initiated by this autonomous process. Also we only regard transmission prior to control: we will consider tracing effective if *R^pre^*<1.

## The Model

**Figure 1:**
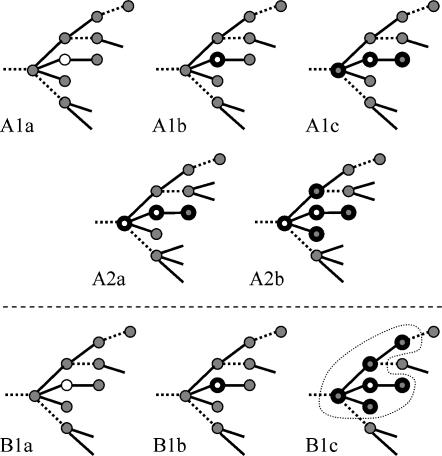
Single-step and iterative tracing on an epidemic tree, developing from left to right. Nodes are infecteds, lines are contacts, contactees that were not infected are not represented on the tree. Grey infecteds are asymptomatic, white infecteds are symptomatic, infecteds with a thick border are isolated or quarantined. Solid lines are traceable contacts, dotted lines are untraceable contacts. A. Single-step tracing. In A1a-c, a symptomatic infected is isolated and his traceable contactees are quarantined. In A2a-b (some time later), one of the quarantined infecteds got symptomatic and his traceable contactees are quarantined. B. Iterative tracing. In B1a-c, a symptomatic infected is isolated and all infecteds directly or indirectly linked to this infected by traceable contacts are quarantined. All quarantined infecteds form a traceable cluster.

We study an epidemic in its initial phase and use a branching process for its description, which means that the epidemic can be regarded as a growing tree. Each branch (contact) connects two nodes (contactees) with a hierarchical relation, the *infector* having infected the *infectee*. If one of the contactees is notified as being infected, by becoming symptomatic or by being traced via a secondary contact, the contact is traced with a probability *p_c_* (all parameters and functions of the model are given in Table 1). Each contact may be traced only once, either from infector to infectee (forwards tracing) or from infectee to infector (backwards tracing), so its traceability can be determined at the time of transmission. Thus, infection trees with traceable and untraceable contacts arise (Figure 1).

**Table 1:**
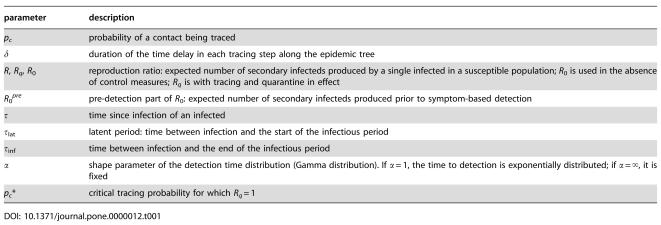
List of most important parameters.

parameter	description
*p_c_*	probability of a contact being traced
δ	duration of the time delay in each tracing step along the epidemic tree
*R*, *R_q_*, *R*_0_	reproduction ratio: expected number of secondary infecteds produced by a single infected in a susceptible population; *R*_0_ is used in the absence of control measures; *R_q_* is with tracing and quarantine in effect
*R*_0_ *^pre^*	pre-detection part of *R*_0_: expected number of secondary infecteds produced prior to symptom-based detection
τ	time since infection of an infected
τ_lat_	latent period: time between infection and the start of the infectious period
τ_inf_	time between infection and the end of the infectious period
α	shape parameter of the detection time distribution (Gamma distribution). If α = 1, the time to detection is exponentially distributed; if α = ∞, it is fixed
p_c_*	critical tracing probability for which *R_q_* = 1

On these infection trees, two types of contact tracing are distinguished. The first type is single-step contact tracing, in which all traceable contactees of a symptomatic case are quarantined, but tracing only continues when an undiscovered infected, quarantined in this way, is detected itself (Figure 1A). The second type is iterative tracing, in which tracing of traceable contactees is continued, and a whole *cluster* of infecteds that is linked through traceable contacts is quarantined (Figure 1B). Such continuation would be possible if a test were available to determine the infection status of traced contactees.

The underlying model for infection dynamics is based on the framework of [4]. In our model, τ measures time since infection of an individual, which starts with a latent period until τ = τ_lat_ without transmission of the pathogen. During the infectious period, lasting from τ_lat_ to τ_inf_, infecteds give rise to β new infecteds per unit of time, as long as they are not detected. As we are interested in the effectiveness of tracing only, we do not model possible transmission that might occur while being isolated, so transmission ‘ceases’ after detection of the infected. This leaves us with the basic reproduction ratio prior to detection, defined as the expected number of secondary infections per infected in a susceptible population before detection, *R*_0_ *^pre^*. Here we have adjusted the interpretation of the model in [4], where *R*_0_ is divided in an asymptomatic and a symptomatic part, by adding a detection delay after becoming symptomatic. The part *R*_0_ *^pre^* is determined by τ_lat_, τ_inf_, β, and the distribution of the detection time, a Gamma-distribution with mean 1 (so time is measured relative to the mean detection time) and shape parameter α. Throughout our analyses, β will be scaled accordingly to achieve a desired value of *R*_0_ *^pre^*. This model construction allows a flexible way of exploring different assumptions about the time to detection, the infectious period, and their overlap, and it enables us to evaluate tracing effectiveness for most infections.

For full understanding of contact tracing in our model, we analyzed four special cases regarding the infectious period and the detection time distribution. The infectious period was assumed to be either very short (all transmission occurs instantaneously, so τ_inf_ = τ_lat_) or very long (of infinite duration, so τ_inf_ = ∞). (In our model, infinite duration can be assumed because each infected will stop spreading the infection after detection. If some infecteds would never be detected, some large τ_inf_<∞ should be taken.) The detection time was assumed to be either fixed (α = ∞) or highly variable (α = 1, i.e. exponentially distributed). As a control we analyzed intermediate cases (results not shown) and four examples of real infections (influenza, SARS, smallpox, and foot-and-mouth disease), of which the parameter values are listed in Table 2. These parameter values were obtained from literature [2], [3], [12]–[14], assuming that the time to detection consists of the incubation period (time to symptom onset) plus a symptom-to-detection delay, which we assumed to be distributed as observed in the SARS epidemic (average 3.67 days, [2]).

**Table 2:**
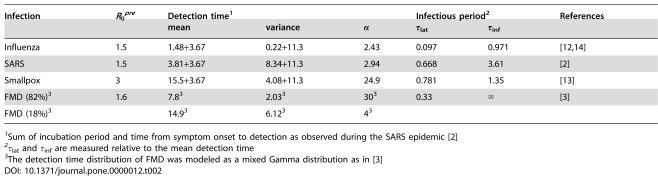
Parameter values for the real infections

Infection	*R*_0_ *^pre^*	Detection time^1^			Infectious period^2^		References
		mean	variance	α	τ_lat_	τ_inf_	
Influenza	1.5	1.48+3.67	0.22+11.3	2.43	0.097	0.971	[Bibr pone.0000012-Group1] 12,14
SARS	1.5	3.81+3.67	8.34+11.3	2.94	0.668	3.61	[2]
Smallpox	3	15.5+3.67	4.08+11.3	24.9	0.781	1.35	[13]
FMD (82%)^3^	1.6	7.8^3^	2.03^3^	30^3^	0.33	∞	[3]
FMD (18%)^3^		14.9^3^	6.12^3^	4^3^			

1 Sum of incubation period and time from symptom onset to detection as observed during the SARS epidemic [2]

2 τ_lat_ and τ_inf_ are measured relative to the mean detection time

3 The detection time distribution of FMD was modeled as a mixed Gamma distribution as in [3]

In our analyses tracing effectiveness will be expressed as the critical tracing probability p_c_*, defined as the proportion of contacts that need to be traced to achieve *R^pre^* = 1. If at least that many contacts are traced, epidemics will certainly die out if transmission during isolation or quarantine is prevented or limited to a small number of health-care workers that do not re-introduce the infection into the general community (see also [5]). If such re-introductions cannot be excluded, a lower *R^pre^* may be aimed for. Because of this threshold of 1, the *R*_0_ *^pre^* values (without tracing) were assumed to have some value larger than 1 (otherwise tracing would not be needed at all) and less than published *R*_0_ values for the specific cases (Table 2). First we study p_c_* as a function of *R*_0_ *^pre^* and τ_lat_ (with tracing delay δ = 0), and second as a function of τ_lat_ and δ (with *R*_0_ *^pre^* = 1.5).

## Results

### Single-step tracing without delays

In an epidemic where single-step tracing is applied, asymptomatic infecteds can spread the infection until they are detected and isolated. Detected infecteds are asked to name their contactees, and a proportion *p_c_* of all contactees will be reported and quarantined. Only when quarantined infecteds are detected themselves, tracing is continued (Figure 1A).

**Figure 2:**
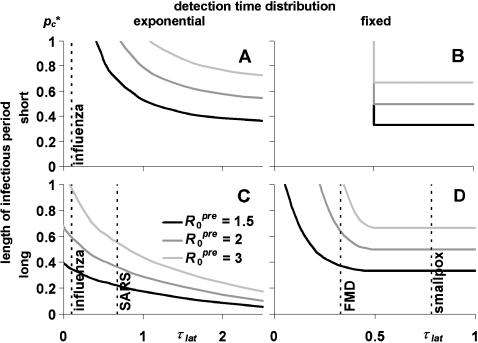
The effectiveness of single-step contact tracing without tracing delays. Effectiveness is expressed as the minimum proportion of contacts that need to be traced for effective control (critical tracing probability p_c_*). The plots show p_c_* as a function of the latent period relative to the mean time to detection (τ_lat_). There are four special cases: A. Short infectious period and variable time to detection; B. Short infectious period and fixed detection time; C. Long infectious period and variable time to detection; and D. Long infectious period and fixed detection time. The three curves denote p_c_* for different values of the pre-isolation reproduction ratio *R*_0_ *^pre^*. Indicated by dashed lines are the average τ_lat_ for four infections, in the panels with closest correspondence to the actual parameter values (Table 2). Influenza appears in two panels with long and short infectious period, because it corresponds to both parameter sets equally.

We determined the critical tracing probability p_c_* as a function of the latent period for three values of *R*_0_ *^pre^* (1.5, 2, and 3). The results are shown in Figure 2. If the detection time is fixed (α = ∞), a too large latent period (larger than the detection time) results in a situation where every infected is detected before transmitting the infection, so tracing need not prevent any transmission; hence the maximum τ_lat_ of 1 in panels 2B and 2D. If we locate the approximate position of real infections (parameter values in Table 2) in Figure 2, we observe that the long infectious period will be the best approximation for most infections, because we only regard pre-detection transmission and infecteds will often still be infectious at the time of detection. This does not entirely hold for influenza, which therefore fits best between the short and long infectious period. Because of the detection period distributions, smallpox and FMD are best described by panel 2D, whereas SARS and influenza fit best into panel 2C (influenza also into 2A).

In three special cases (panels 2A,B,D), with fixed incubation period and/or short infectious period, the proportion of contacts to be traced is at least 1−1/*R*_0_ *^pre^*. This lower limit 1−1/*R*_0_ *^pre^* is due to forwards tracing, when all infecteds that are traceable via their infector, are quarantined before the end of their latent period. Then, the effective reproduction ratio will be equal to the untraceable proportion of *R*_0_ *^pre^*, i.e. *R_q_* = (1−*p_c_*)*R*_0_ *^pre^*, resulting in p_c_* = 1−1/*R*_0_ *^pre^*. This is likely to be case with smallpox (panel 2D). However, if the latent period is short, as seen for influenza and possibly FMD, quarantine will occur too late to prevent all infections and more contacts need to be traced.

In the fourth panel (2C), with variable detection time and long infectious period, effective contact tracing requires a proportion of contacts *smaller* than 1−1/*R*_0_ *^pre^* to be traced, if the latent period is large enough (like SARS). This can be explained as follows. If the detection time is variable and the latent and infectious periods are large, many infecteds will be detected before becoming infectious. If many infecteds are not infectious before being detected, the few that are should be very infectious (“superspreaders”) to attain a given *R*_0_ *^pre^* (the average number of secondary infections before detection). Because many of the infectees of these superspreaders will be detected early (variable detection time), the superspreaders will be quarantined after backwards tracing, which adds to the effect of forwards tracing preventing infectees to reach their infectious period.

Effectiveness may be very sensitive to the latent period, especially if there is little variation in the detection time. This is most apparent in the sharp transition in panel 2B, where tracing is only effective if all infectors are detected (at τ = 1−τ_lat_) before the infectious period (at τ = τ_lat_), so τ_lat_>0.5. The high sensitivity can be a problem for assessing the tracing effectiveness for a specific infection: the conclusion may largely depend on how correct the available estimates for the latent and incubation periods are, the incubation period determining the time to detection. Figure 2 indicates that this might be a problem for FMD.

### Single-step tracing with delays

In the previous section tracing was an instantaneous process: detection and isolation of a given case were followed by quarantine of traceable contactees of that case without any delay. The effect of a delay of duration δ is that contactees of detected infecteds can continue transmitting the infection for an extra δ time units, unless they are detected themselves during this interval.

**Figure 3:**
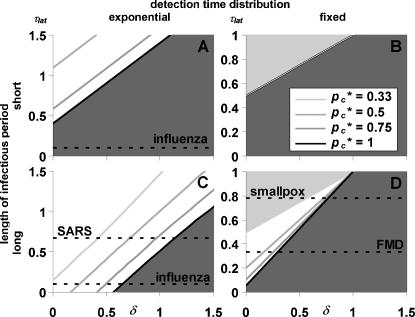
The effectiveness of single-step contact tracing with tracing delays, with the pre-detection reproduction ratio *R*_0_ *^pre^* = 1.5. Effectiveness is expressed as the minimum proportion of contacts that need to be traced for effective control (critical tracing probability p_c_*). The contour plots show p_c_* as a function of the tracing delay δ and the latent period τ_lat_, measured relative to the mean detection time, for four special cases: A. Short infectious period and variable incubation period; B. Short infectious period and fixed incubation period; C. Long infectious period and variable incubation period; and D. Long infectious period and fixed incubation period. Dark grey shadows indicate areas where tracing is ineffective, light grey shadows indicate areas where p_c_* = 0.33. Indicated by dashed lines are the average τ_lat_ for four infections, in the panels with closest correspondence to the actual parameter values (Table 2). Influenza appears in two panels with long and short infectious period, because it corresponds to both parameter sets equally.

The effect of a delay in each tracing step was studied by determining the critical tracing probability p_c_* for *R*_0_ *^pre^* = 1.5, as a function of the latent period and tracing delay. Figure 3 shows the resulting contour plots for the four special cases with single-step tracing, and it indicates the approximate positions of the four real infections. Along the τ_lat_ axis (δ = 0) lie the *R*_0_ *^pre^* = 1.5 curves of Figure 2.

**Figure 4:**
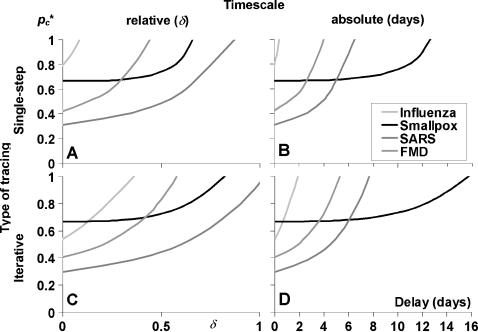
The effectiveness of single-step and iterative contact tracing for control of influenza, smallpox, SARS, and foot-and-mouth disease. Effectiveness is expressed as the minimum proportion of contacts that need to be traced for effective control (critical tracing probability p_c_*); p_c_* is plotted as a function of the relative delay (δ, proportion of the incubation period) or the absolute delay (days).

It appears that the iso-p_c_* contours in Figure 3 are linear in three of the four special cases (panel 3A,B,D), and approximately linear in the fourth case, with long infectious period and variable incubation time (panel 3C: τ_inf_ = ∞; α = 1). The slopes of all (approximate) lines are always between 0.5 and 1.5. This means that the effect of tracing delays is comparable to the sensitivity to the latent period as observed in Figure 2, so plots of the critical tracing probability as a function of the delay will resemble the plots in Figure 2, only mirrored (as in Figure 4).

In the contour plots in Figure 3, tracing is ineffective in the dark grey areas (small latent period or large delay), so smallpox and SARS control are predicted to be able to cope with some delays, whereas it might be more of a problem with FMD and influenza. If forwards tracing is maximally effective and not affected by the delay, p_c_* = 1−1/*R*_0_ *^pre^* = 0.33 as indicated by the light grey area (smallpox if δ<0.5). If the detection time is variable, tracing may be effective even if a proportion less than 1−1/*R*_0_ *^pre^* is traced (SARS if δ<0.4, panel 3C). Because backwards tracing is needed to attain this result, it is only possible when the tracing delay is shorter than the infectious period (not shown in the Figures).

The contour plots show that for some combinations of latent period and delay, the sensitivity to small changes in the delay is large (contours lie close to one another). Then, small changes in the delay can have a major impact when the time of quarantine is shifted from just before to just after the end of the latent period, similar to the sensitivity to τ_lat_ as observed in Figure 2. This effect is most dramatic if there is little variation in the detection time (smallpox: δ≈0.7, and FMD: δ≈0.2).

Figure 4 shows the effectiveness of single-step tracing for SARS, smallpox, influenza, and FMD using the parameter values listed in Table 2. Panel 4A shows the relation between p_c_* and the relative tracing delay δ, from which it appears that the general cases shown in Figures 2 and [3] are good predictors for assessing tracing effectiveness. Influenza can be placed somewhere between long and short infectious periods (panels 2A,C, and 3A,C): it is hardly effective without delay, and ineffective already if δ = 0.1. SARS control requires a tracing probability p_c_*<1−1/*R*_0_ *^pre^* which is relatively insensitive to delays. Smallpox requires p_c_* = 1−1/*R*_0_ *^pre^*, and is insensitive to delays up to some point (δ≈0.6) where tracing becomes quickly ineffective. Finally, FMD can be traced effectively only if δ is small, but it is sensitive to delays already if δ is small and requires a tracing probability p_c_*>1−1/*R*_0_ *^pre^*. Measured in real time (panel 4B), effectiveness of tracing appears to be highly dependent on the actual generation time of the infection. Influenza control is hardly possible, FMD control will be difficult, whereas tracing is likely to be successful for smallpox and SARS.

### Iterative tracing

Suppose a test is available to determine the infection status of traced contactees. We can then continue tracing iteratively before infected contactees are detected due to symptoms, until no further infecteds are found (Figure 1B).

We evaluated iterative tracing with and without delays for the same cases as single-step tracing, resulting in Figures similar to Figures 2 and [3] (see Supporting Information). As expected, the universal effect of iterative tracing is to lower p_c_*, although the lower limits 1−1/*R*_0_ *^pre^* as observed in three of the four special cases remain intact. The largest difference between single-step and iterative tracing is observed when single-step tracing is on the brink of being effective, so that is the only situation where iterative tracing will make a difference (see also Figure 4). Without delays this difference is rather intuitive: single-step tracing is not effective if the latent period is small, but iterative tracing will always be effective, because if all contacts are traceable, the first detection is immediately followed by quarantine of every infected. This is the case with influenza, although the improvement will probably not be sufficient to making tracing effective for influenza control. On the other hand, when the latent period is large and single-step tracing is effective, iterative tracing only improves on this if there are significant delays, providing one or two more days to reach the required p_c_* (smallpox, SARS, and FMD).

## Discussion

We studied the effectiveness of contact tracing in a model for the start of an epidemic, that is flexible enough to use for most infections. Effectiveness of contact tracing was expressed as the minimum proportion of contacts that need to be traced to obtain a reproduction ratio prior to control of 1 (p_c_*). Other threshold values may be chosen if more is known about transmission of the infection to the general community while infectious individuals are being isolated or quarantined. For instance, if isolated infecteds still cause an average of 0.3 new cases in the general community after isolation or quarantine, p_c_* should be redefined such that *R^pre^* = 0.7.

The first conclusion from our model is that, for a given *R*_0_ *^pre^*, the critical tracing probability can take any value depending on all infectious disease characteristics in the model: the latent period, the infectious period, and the detection time distribution. In contrast to some earlier publications on contact tracing [7], [9], there exists no general expression for p_c_* as there is for the proportion to vaccinate for effective control in a well-mixed population. For smallpox the relation p_c_* = 1−1/*R*_0_ *^pre^* holds reasonably well, but for SARS it is smaller, and for influenza and FMD it is larger.

The second conclusion is that a variable detection time improves tracing effectiveness, possibly even resulting in p_c_*<1−1/*R*_0_ *^pre^*. This does not mean, of course, that one should aim at late detection of some infecteds (which would increase *R*_0_ *^pre^*), but that apparent variability is an argument to use tracing. The reason is that the few infecteds that are detected late (or not at all, which is essentially the same) will be discovered by backwards tracing which is an additional effect to forwards tracing that in itself may already result in p_c_* = 1−1/*R*_0_ *^pre^*. It was earlier found that p_c_* can be smaller than 1−1/*R*_0_ *^pre^* [9], but by a different mechanism, namely the presence of shared contacts in a network.

The third conclusion is that the sensitivity of tracing effectiveness to the latent period and tracing delay may be large, especially in the case of single-step tracing. If this is the case already with a small delay (influenza and to a lesser extent FMD), reliability of parameter estimates will be crucial to establish whether tracing is advantageous. If it is only the case with larger delays (smallpox, SARS), tracing may be effective as long as it is done quick enough.

The fourth conclusion is that in most situations single-step and iterative tracing are almost equally effective. A considerable difference can only be expected if single-step tracing is not or hardly effective, which is also when the sensitivity to the latent period and tracing delay is largest (see above). Thus, influenza control will benefit from iterative tracing already without delays, whereas with the other infections one or two days may be gained (Figure 4C,D), thus having more time to achieve the required p_c_*. In the real world, the choice between single-step and iterative tracing will be based on what is possible. Capacity problems may reduce the effectiveness of iterative tracing if effort is directed towards secondary contactees prior to primary contactees; on the other hand, if not quarantine but vaccination is applied, it might be worthwhile to traced contactees of contactees even without diagnostic tests [6].

For determining the cost-effectiveness of contact tracing, not only the critical tracing probability p_c_* is required, but also how easily that p_c_* can be achieved. A key aspect will be the possibility to distinguish relevant contacts: for control of sexually transmitted diseases relevant contacts are easily identified (that is, if people are willing to co-operate), but in case of respiratory pathogens like SARS or influenza the notion of relevant contacts is rather diffuse. Good insight will be obtained by not only regarding the sensitivity of tracing (*p_c_*), but also the *positive predictive value*: what proportion of traced individuals is actually infected, and if we put more effort into increasing *p_c_*, how will it decrease the positive predictive value?

A relatively easy way to increase the proportion of traced contacts for respiratory pathogens like influenza might be quarantine of households. This can be effective if households are the prime location for spread of the infection; specific models taking into account the contact structures within and between households will be better-suited to study this strategy.

In our model we only regarded transmission of the infection before tracing or isolation, allowing us to focus on the characteristics of the contact tracing itself. Regarding the control of animal disease epidemics, e.g. foot-and-mouth disease or avian influenza, it is not unreasonable to assume that there will be no other transmission: all infected and suspected farms will be culled and they really will stop being infectious. However, with human infections it is very likely that isolation and quarantine will be less effective. This results in a complicated situation, because transmission will not only be reduced after detection, the contact structure will also change, with only a limited number of people (health-care workers) having contact with multiple isolated patients. For the case of SARS, a more detailed model on exactly this aspect was studied by Lloyd-Smith et al [5], who indeed concluded that minimizing within-hospital transmission might be crucial, especially if tracing and isolation occur rather slowly. Further studies are required for this complex problem. The present paper provides a good understanding of the principles of contact tracing, and how infectious disease characteristics determine the effectiveness.

## Methods

The effect of single-step tracing can be measured by the effective reproduction ratio *R_q_*, the average number of new infections per infected. Whereas the rate of being traced backwards at time τ since infection (due to detection of any infectee) is equal for all infecteds, the rate of being traced forwards (due to detection of the infector) depends on the generation-time distribution, which in turn depends on the number of traceable generations backwards in the transmission tree (traceable ancestors). Hence, infectives need to be typed according to the number of traceable ancestors *j*, and *R_q_* is the largest eigenvalue of the next-generation matrix *K* with entries *k_ij_* being the expected number of type-*i* infecteds (with *i* traceable ancestors) produced per type-*j* infected [15]. Because type-*j* infecteds only produce type-0 and type-*j*+1 infecteds, by untraceable and traceable contacts respectively, all entries other than *k*_0*j*_ and *k_j_*_+1,*j*_ are equal to 0.

Even though the matrix is infinitely large, we conjecture from all observed numerical calculations that the positive elements *k_j_*_+1,*j*_ converge in the sense that |*k_j_*_+1,*j*_−*k_j_*_+2,*j*+1_|→0 as *j*→∞. Therefore, for numerical evaluation we ‘closed’ the matrix to an (*n*+1)^2^ matrix with *k_nn_* = *k_n_*_+1,*n*_. For the four special cases, *R_q_* could be calculated numerically in Mathematica®, but for other cases considered, the entries of the matrix had to be determined by numerical simulation. Analysis of single-step tracing with delay could be done by calculating *R* from the next-generation matrix, as for the model without delay (see Supporting Information for all details).

With iterative tracing, isolation of a single infected results in quarantine of a cluster of infecteds, all mutually linked by traceable contacts (Figure 1B). By recognizing these clusters of infectives, an epidemic of infected individuals can be regarded as an epidemic of traceable clusters. Each untraceable contact in the transmission tree results in infection of a new cluster index case, so the average number of untraceable contact infections caused by a single cluster, the cluster reproduction ratio *R_c_*, determines the effectiveness of iterative contact tracing. If *Y*(*p_c_*) denotes the expected cumulative infectiousness of a cluster at the time of cluster quarantine, then
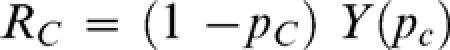
1


As with single-step tracing, effectiveness of iterative tracing is determined by considering the critical tracing probability (for achieving *R_c_* = 1):
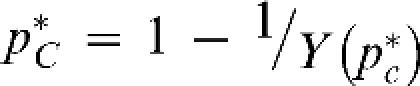
2If *p_c_* = 0, then each infective will be a separate cluster, and *R_c_* = *Y*(0) = *R*_0_ *^pre^* by definition. For *p_c_*>0 the cluster size will be larger than one, but cluster infectiousness need not be larger, as backwards tracing can reduce the infectious period of superspreaders with large detection time as it does in single-step tracing.

For two of the four special cases (α = ∞), and for the case considered by Müller et al [7] (α = 1, τ_inf_ = ∞, τ_lat_ = 0), *Y*(*p_c_*) can be calculated numerically in Mathematica®. For all other cases, including the real infections, stochastic simulations were needed.

Incorporating delays into iterative tracing does not generally change the concept of *R_c_*, but it makes the calculation of *Y*(*p_c_*) very complicated, because there is no longer a single time of cluster quarantine. Two complications arise: first, delays may cause contactees in the chain emanating from a symptomatic infected to become symptomatic themselves before the tracing process reaches them. They then initiate a new tracing process of their own within the same cluster. Second, if the infection process is faster than the tracing process, the cluster size grows infinitely large and iterative tracing becomes ineffective. We determined p_c_* by stochastic simulation of clusters until quarantine of the final infected (see Supporting Information for all details).

## Supporting Information

Supporting Information Supporting information for the paper “The Effectiveness of Contact Tracing in Emerging Epidemics”
(0.60 MB PDF)
