# Supplementary material for: The Effectiveness of Contact Tracing in Emerging Epidemics
Source: PLoS One. 2006 Dec 20;1(1):e12. doi: 10.1371/journal.pone.0000012 (PMC1762362; doi:10.1371/journal.pone.0000012)

Supporting information

with the paper "The effectiveness of contact tracing in emerging epidemics"

by D. Klinkenberg, C. Fraser, and J.A.P. Heesterbeek

A. Single-step tracing

For calculation of the reproduction number R with single-step tracing, we require the elements k_{ij} of the next-generation matrix

$$\begin{pmatrix} k_{00} & k_{01} & k_{02} & \cdots & k_{0n} \\ k_{10} & 0 & 0 & \cdots & 0 \\ 0 & k_{21} & 0 & \cdots & 0 \\ \vdots & \vdots & \ddots & \ddots & 0 \\ 0 & 0 & 0 & k_{n,n-1} & k_{nn} \end{pmatrix}$$

In the matrix, k_{ij} is the expected number of type-i infecteds produced by one type-j infected, where an infected of type j is someone with exactly j traceable contacts backwards in the transmission tree (traceable ancestors). For instance, infecteds of type 1 were infected through a traceable contact by their infector, who in turn were infected through an untraceable contact. Type-1 infecteds produce (on average) k_{21} type-2 infecteds through traceable contacts, and k_{01} type-0 infecteds through untraceable contacts. Thus, an infinite-size next-generation matrix is obtained which can be closed to an $(n+1)^2$ matrix as above by choosing n large enough and conjecturing that $|k_{j+1,j} - k_{j+2,j+1}| \to 0$ as $j \to \infty$.

R can be calculated numerically in Mathematica® for the four special cases discussed in the main text, which will be shown in the next sections. For all other

cases, simulations were used to determine the first few elements, as was done for the real infections considered in the paper.

A1. Single-step tracing if $\alpha = 1$ and $\tau_{inf} = \tau_{lat}$

If we denote by $\widetilde{\beta}$ the average number of secondary infections caused at $\tau = \tau_{lat}$, given that the infected is not yet isolated or quarantined, then $R_0^{pre} = e^{-\tau_{lat}}\widetilde{\beta}$, where $e^{-\tau_{lat}}$ is the probability that the infected is not isolated by $\tau = \tau_{lat}$.

Because of the exponential distribution of the incubation period, the hazard of being quarantined through the infector is time-independent. Therefore, the expected number of infecteds produced by type-j infecteds are equal for all $j > 0$, and a 2×2 matrix will suffice for calculation of R.

The element k_{10} denotes the number of traceable infectees, produced by an infected that was itself produced via an untraceable contact:

$$k_{10} = p_c R_0^{pre}$$

which is not affected by contact tracing and quarantine at all, because the instantaneous production of all infectees ($\tau_{inf} = \tau_{lat}$) prevents backwards tracing to reduce transmission.

The element k_{11} denotes the number of traceable infectees, produced by an infected that was itself produced via a traceable contact:

$$k_{11} = e^{-\tau_{lat}} p_c R_0^{pre},$$

in which $e^{-\tau_{lat}}$ is the probability that the infector is detected before the end of the latent period.

We use the identity $(1-p_c)\, k_{j+1,j} = p_c\, k_{0j}$ to calculate k_{00} and k_{01}, and subsequently determine the largest eigenvalue of the next-generation matrix to obtain the reproduction ratio

$$R = R_0^{pre}\left(1 - p_c + p_c e^{-\tau_{lat}}\right)$$

and the critical tracing probability ($R = 1$)

$$p_c{}^* = \frac{R_0^{pre} - 1}{R_0^{pre}\left(1 - e^{-\tau_{lat}}\right)}.$$

Adding a tracing delay of duration $\delta < \tau_{lat}$ changes k_{11} into $k_{11} = e^{-(\tau_{lat}-\delta)} p_c R_0^{pre}$, which results in the following R and $p_c{}^*$:

$$R = R_0^{pre}\left(1 - p_c + p_c e^{-(\tau_{lat}-\delta)}\right)$$
$$p_c{}^* = \frac{R_0^{pre} - 1}{R_0^{pre}\left(1 - e^{-(\tau_{lat}-\delta)}\right)}$$

If $\delta \geq \tau_{lat}$, tracing is ineffective.

A2. Single-step tracing if $\alpha = \infty$ and $\tau_{inf} = \tau_{lat}$

If we denote by $\tilde{\beta}$ the average number of secondary infections produced at $\tau = \tau_{lat}$, then $R_0^{pre} = \tilde{\beta}$ if $\tau_{lat} < 1$ (in the absence of contact tracing); otherwise $R_0^{pre} = 0$, and the model is not defined for $R_0^{pre} > 0$.

Because the detection time is fixed and all secondary infections are produced instantaneously, tracing and quarantine will not be effective if infections are produced before detection of the infector, which is if $\tau_{lat} \leq 0.5$.

If $\tau_{lat} > 0.5$ on the other hand, type-1 infecteds will not produce any new infection, so the only relevant element in the transmission matrix is $k_{00} = (1\text{-}p_c)R_0^{pre}$, resulting in the reproduction ratio

$$R = (1 - p_c)R_0^{pre},$$

and the critical tracing probability

$$p_c^* = 1 - 1/R_0^{pre}.$$

With a tracing delay δ, the same relation holds for $\tau_{lat} > (1 + \delta)/2$.

A3. Single-step tracing if $\alpha = 1$ and $\tau_{inf} = \infty$

If an infected is not isolated before τ_{lat}, the expected duration of the infectious period (from τ_{lat} to isolation) is 1, so $R_0^{pre} = e^{-\tau_{lat}}\beta$, where $e^{-\tau_{lat}}$ is the probability not to be isolated before the start of the infectious period, and β is the infection rate.

Because of the exponential distribution of the time to detection, the processes of quarantine and isolation are Markov processes. Therefore, the rate at which infecteds are traced trough their infector is equal for all $j > 0$, and a 2×2 matrix will suffice for calculation of R.

We first consider the elements k_{10} and k_{11} for $\tau_{lat} = 0$. The probability $P_x^{(0)}$ for a type-0 infected to produce x traceable infectees is

$$P_x^{(0)} = \frac{(p_c\beta)^x (x+1)}{\prod_{z=0}^{x}(1+z+p_c\beta)},$$

which is the probability that the first x events are infection events, and the $x+1^{\text{st}}$ event is an isolation/quarantine event. Because of the Markov property of the isolation/quarantine process, the probabilities for each next event are due to competing hazards; each next infection increases the isolation/quarantine hazard by 1 whereas the infection hazard remains constant at $p_c\beta$.

The probability $P_x^{(0)}$ is used to calculate k_{10} as

$$k_{10} = \sum_{x=0}^{\infty} xP_x^{(0)} = \sum_{x=0}^{\infty} x\frac{(p_c\beta)^x (x+1)}{\prod_{z=0}^{x}(1+z+p_c\beta)}$$

Similarly, k_{11} is equal to

$$k_{11} = \sum_{x=0}^{\infty} x\frac{(p_c\beta)^x (x+2)}{\prod_{z=0}^{x}(2+z+p_c\beta)},$$

where ‘1’ is replaced by ‘2’ because of the possibility of being traced through the infector. The identity (1-p_c) $k_{j+1,j} = p_c\, k_{0j}$ is used to calculate k_{00} and k_{01}.

Incorporation of a latent period into this framework is easy, because the number of secondary cases will be equal to the expressions above, *given* that the infected is not quarantined or isolated during the latent period. Therefore, an adjustment needs to be made by multiplication by the probability to reach the end of the latent period without being isolated or quarantined:

$$k_{10} = e^{-\tau_{lat}} \sum_{x=0}^{\infty} x \frac{(p_c\beta)^x (x+1)}{\prod_{z=0}^{x} (1+z+p_c\beta)}$$

$$k_{11} = e^{-2\tau_{lat}} \sum_{x=0}^{\infty} x \frac{(p_c\beta)^x (x+2)}{\prod_{z=0}^{x} (2+z+p_c\beta)}$$

A numerical evaluation of k_{10} and k_{11} was done to calculate R and determine p_c*.

A tracing delay of duration δ does not change $P_x^{(0)}$, but it does change k_{10}:

$$k_{10} = e^{-\tau_{lat}} \sum_{x=0}^{\infty} \left(x + p_c\beta\left(1-e^{-\delta}\right)\frac{x}{x+1} \right) \frac{(p_c\beta)^x (x+1)}{\prod_{z=0}^{x} (1+z+p_c\beta)}.$$

Here, $p_c\beta\left(1-e^{-\delta}\right)\frac{x}{x+1}$ is an extra amount of expected infectiousness for a duration δ ($p_c\, \beta\, (1 - e^{-\delta})$) given that the last event was isolation of a contactee and not of the infected itself (with probability $x/(x+1)$).

For the element k_{11}, we also need to take into account the possibility that the infector is detected before τ_{lat}, but the tracing step is completed after τ_{lat}. Calculation of the extra infectiousness term depends on whether δ is larger or smaller than τ_{lat}.

Let $\delta < \tau_{lat}$ and T_s ($\tau_{lat} - \delta < T_s < \tau_{lat}$) be the (stochastic) time that the infector is detected and isolated (quarantine will take place at $T = T_s + \delta$). The probability density function $f(\tau_s)$ is

$$f(\tau_s) = \tfrac{1}{1-e^{-\delta}} e^{-(\tau_s - \tau_{lat} + \delta)}$$

The expected number of traceable infectees X, given that the infector is symptomatic at $T_s = \tau_s$, is

$$E(X | T_s = \tau_s) = \beta p_c \int_{\tau_{lat}}^{\tau_s + \delta} e^{-(\sigma - \tau_{lat})} d\sigma = \beta p_c \left(1 - e^{-(\tau_s - \tau_{lat} + \delta)}\right).$$

Thus, the expected number of traceable infectees X, given that $\tau_{lat} - \delta < T_s < \tau_{lat}$, is

$$E(X | \tau_{lat} - \delta < T_s < \tau_{lat}) =$$

$$\int_{\tau_{lat} - \delta}^{\tau_{lat}} \tfrac{1}{1-e^{-\delta}} e^{-(\tau_s - \tau_{lat} + \delta)} \beta p_c \left(1 - e^{-(\tau_s - \tau_{lat} + \delta)}\right) d\tau_s =$$

$$\tfrac{1}{2} \beta p_c \left(1 - e^{-\delta}\right)$$

A similar calculation can be made for $\delta \geq \tau_{lat}$, which in the end results in

$$k_{11} = e^{-2\tau_{lat}} \left(e^{\delta} - 1\right) \times \tfrac{1}{2} \beta p_c \left(1 - e^{-\delta}\right) +$$

$$e^{-2\tau_{lat}} \sum_{x=0}^{\infty} \left(x + p_c \beta \left(1 - e^{-\delta}\right) \frac{x+1}{x+2} \right) \frac{(p_c \beta)^x (x+1)}{\prod_{z=0}^{x} (1 + z + p_c \beta)} \qquad \forall \delta \leq \tau_{lat}$$

$$k_{11} = e^{-2\tau_{lat}}\left(e^{\tau_{lat}} - 1\right)\times \beta p_c\left(1 - \tfrac{1}{2}e^{-\delta}\frac{e^{\tau_{lat}} - e^{-\tau_{lat}}}{1 - e^{-\tau_{lat}}}\right) +$$

$$e^{-2\tau_{lat}}\sum_{x=0}^{\infty}\left(x + p_c\beta\left(1 - e^{-\delta}\right)\frac{x+1}{x+2}\right)\frac{\left(p_c\beta\right)^x (x+1)}{\prod_{z=0}^{x}\left(1 + z + p_c\beta\right)} \qquad \forall \delta > \tau_{lat}$$

Here, $e^{-2\tau_{lat}}\left(e^{\delta} - 1\right)$ and $e^{-2\tau_{lat}}\left(e^{\tau_{lat}} - 1\right)$ are the probabilities that the infector gets symptomatic during the latent period, and the infected itself does not.

A4. Single-step tracing if $\alpha = \infty$ and $\tau_{inf} = \infty$

The incubation period is fixed at 1, so each (untraced) infected transmits the infection from $\tau = \tau_{lat}$ to $\tau = 1$, resulting in $R_0^{pre} = (1 - \tau_{lat})\beta$.

By $\beta^{(j)}(\tau)$ we denote the expected infectiousness of a type-j infected at time τ after infection (of the type-j infected itself). By $q^{(j)}(\tau), j > 0$ we denote the probability density of a type-j infected being quarantined. A series of subsequent $\beta^{(j)}(\tau)$ and $q^{(j)}(\tau)$ can thus be obtained:

$$\beta^{(0)}(\tau) = 0 \forall 0 \le \tau < \tau_{lat}$$
$$\beta^{(0)}(\tau) = \beta \forall \tau_{lat} \le \tau \le 1$$

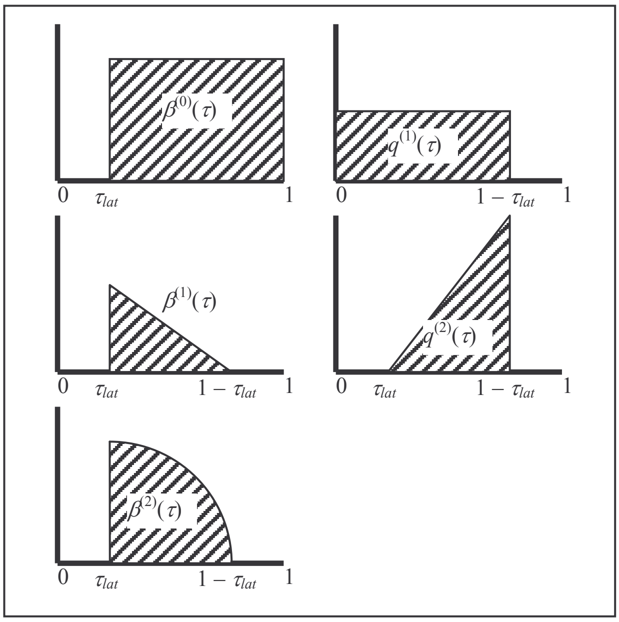

$$q^{(j)}(\tau) = \frac{\beta^{(j-1)}(1-\tau)}{\int_0^1 \beta^{(j-1)}(\sigma)d\sigma}$$

$$\beta^{(j)}(\tau) = 0 \forall 0 \le \tau < \tau_{lat}$$
$$\beta^{(j)}(\tau) = \beta\left(1 - \int_0^{\tau} q^{(j)}(\sigma)d\sigma\right) \forall \tau_{lat} \le \tau \le 1$$

All elements $k_{j+1,j}$ and k_{0j} can be obtained by calculating the area under $\beta^{(j)}(\tau)$ and multiplying by p_c and $1 - p_c$ respectively. By calculation of the elements for $j = 0, 1, \ldots, n$ an $(n+1)^2$ next-generation matrix is obtained, from which R can be obtained as its largest eigenvalue. We did so for $n = 8$, because we considered $| k_{9,8} - k_{10,9} | \leq 0.00006\beta p_c$ small enough.

Tracing delays do not affect $\beta^{(0)}(\tau)$, but $q^{(j)}(\tau)$ and $\beta^{(j)}(\tau)$ change. The sketches below make clear what happens if $\delta \leq \tau_{lat}$ or if $\delta > \tau_{lat}$. In the latter case, the area under $q^{(j)}(\tau)$ is smaller than 1:

$$q^{(j)}(\tau) = 0 \forall 0 \leq \tau < \delta$$
$$q^{(j)}(\tau) = \frac{\beta^{(j-1)}(1-\tau+\delta)}{\int_0^1 \beta^{(j-1)}(\sigma)d\sigma} \forall \delta \leq \tau \leq 1$$

$$\beta^{(j)}(\tau) = 0 \forall 0 \leq \tau < \tau_{lat}$$
$$\beta^{(j)}(\tau) = \beta\left(1 - \int_0^\tau q^{(j)}(\sigma)d\sigma\right) \forall \tau_{lat} \leq \tau \leq 1$$

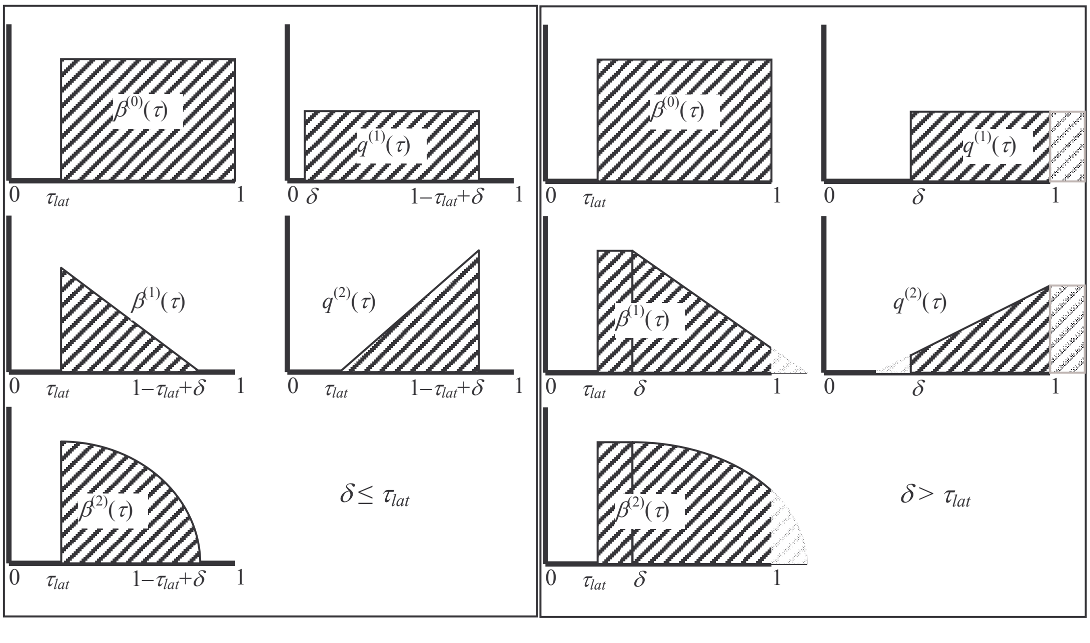

A5. Simulations for single-step tracing

For other cases such as all real infections simulations were carried out to obtain the elements k_{ij}. To this end, we reparameterized the model in the following way:

$$\beta_0 = \beta p_c$$

$$\beta_1 = \beta(1 - p_c),$$

so β_0 is the transmission rate for traceable contacts and β_1 is the transmission rate for untraceable contacts.

We took some values for δ, τ_{lat} and β_0 and we simulated transmission through traceable contacts by type-0 infecteds, by type-1 infecteds, etc. For each type j, $k_{j+1,j}$ was determined and compared with $k_{j,j\text{-}1}$ to check if the matrix could be closed. This was often the case if $j = 3$ or $j = 4$.

Then the identity (1-p_c) $k_{j+1,j} = p_c\, k_{0j}$ was used to determine p_c*. We solved β from $\beta_0 = \beta p_c$*, calculated R_0^{pre}, and a (R_0^{pre}, p_c*) pair was obtained.

By choosing a series of β_0 values, a series of (R_0^{pre}, p_c*) pairs was obtained for one specific case of the model (with some δ, τ_{lat}, and τ_{inf}). Interpolation with the SplineFit function in Mathematica® was used to determine p_c* for any R_0^{pre}.

B. Iterative tracing

For calculation of the cluster reproduction ratio R_c, the cumulative infectiousness of a cluster of infecteds linked by traceable contacts needs to be determined. This

often requires simulation, though in some of the special cases numerical calculation is possible.

B1. Iterative tracing if $\alpha = 1$ and $\tau_{inf} = \tau_{lat}$

In this special case a cluster grows in distinct infection generations until one of the infecteds is isolated, at which time the complete cluster is quarantined. If $\tau_{lat} \to 0$, we see that $p_c^* \to 1$ because clusters will grow very fast and become very large before they are detected. If $\tau_{lat} > 0$, exact calculation of the expected cluster infectivity is not straightforward because of dependencies between sizes of infection generations, cluster infectiousness and the cluster quarantine rate. Therefore, simulations were used to determine p_c^*.

Simulations were carried out as follows. First, the model was reparameterized: $\tilde{\beta}_0 = p_c\tilde{\beta}; \tilde{\beta}_1 = (1 - p_c)\tilde{\beta}$, where the tilde indicates the instantaneous infection process. Then, we chose values for δ, τ_{lat}, and $\tilde{\beta}_0$, and we simulated 100,000 clusters, of which we determined the average infectiousness (a function of $\tilde{\beta}_1$).

From the average cluster infectivity a value for $\tilde{\beta}_1$ was obtained by solving $R_c = 1$. From $\tilde{\beta}_0$ and $\tilde{\beta}_1$, β and p_c^* were calculated to obtain an (R_0^{pre}, p_c^*) pair. After determining a series of (R_0^{pre}, p_c^*) pairs from simulations with different values of $\tilde{\beta}_0$ (while keeping δ and τ_{lat} constant), the SplineFit function in Mathematica® was used for interpolation, so that p_c^* could be determined for any R_0^{pre}.

B2. Iterative tracing if $\alpha = \infty$ and $\tau_{inf} = \tau_{lat}$

In this special case a cluster grows in distinct infection generations until isolation of the cluster index case at $\tau = 1$. The number of infection generations within the

cluster (excluding the cluster index case, generation 0), denoted by n, is equal to the largest integer smaller than $1/\tau_{lat}$. Thus,

$$R_c = \tilde{\beta}_1 \left(\sum_{i=0}^{n-1} \tilde{\beta}_0^{\,i} \right),$$

summed for $i = 0$ to $n - 1$, because the nth generation within the cluster is quarantined before being infectious. By solving $R_c = 1$, p_c* can be obtained for any R_0^{pre} and τ_{lat}.

With a tracing delay $\delta \leq \tau_{lat}$, n is equal to the largest integer smaller than $(1 - \delta)/(\tau_{lat} - \delta)$. If $\delta > \tau_{lat}$, tracing is ineffective.

B3. Iterative tracing if $\alpha = 1$ and $\tau_{inf} = \infty$

In this special case, an exact calculation of R_c can only be done if $\delta = 0$ and $\tau_{lat} = 0$. Then, the dynamics within the cluster is a Markov process, consisting of a series of (traceable) infection events until one isolation event, which is followed by cluster quarantine. The expected number of untraceable contacts originating from the cluster between the i-1st and i^{th} event is equal to the untraceable contact rate × the expected time interval between events = $\beta i(1 - p_c) \times 1/(i + \beta i p_c) = \beta(1 - p_c)/(1 + \beta p_c)$, independent of i. Thus, R_c is equal to this number multiplied by the expected number of events before cluster quarantine:

$$R_c = \frac{\beta(1 - p_c)}{1 + \beta p_c} \times \sum_{x=1}^{\infty} x \frac{(\beta p_c)^{x-1}}{(1 + \beta p_c)^x} = \beta(1 - p_c) = R_0^{pre}(1 - p_c).$$

This result gives $p_c{*} = 1 - 1/R_0^{pre}$, which was also found by Müller et al (2000) for this specific model.

If $\tau_{lat} > 0$ and/or $\delta > 0$, the Markov property is lost, and simulations are required to obtain $p_c{*}$. The procedure is described in Section B5.

B4. Iterative tracing if $\alpha = \infty$ and $\tau_{inf} = \infty$

R_c can be calculated exactly by integration over the expected infectiousness functions $\beta^{(j)}(t)$ at cluster age t for all infection generations j. The cluster is quarantined at $t = 1$, when the cluster index case gets symptomatic:

$$\beta^{(0)}(t) = 0 \forall 0 \le t \le \tau_{lat}$$

$$\beta^{(0)}(t) = \beta(1 - p_c) \forall \tau_{lat} < t \le 1$$

$$\beta^{(j)}(t) = 0 \forall 0 \le t \le (j+1)\tau_{lat}$$

$$\beta^{(j)}(t) = \beta(1 - p_c) \times \int_0^{t-\tau_{lat}} \beta^{(j-1)}(\sigma) d\sigma =$$

$$\beta(1 - p_c) \times \tfrac{1}{j!} (\beta p_c)^j (t - (j+1)\tau_{lat})^j \forall (j+1)\tau_{lat} < t \le 1$$

If a tracing delay $\delta \le \tau_{lat}$ is taken into account, then the above infectiousness functions hold for $t \le 1 + j\delta$ (instead of $t \le 1$). If $\delta > \tau_{lat}$, simulations are required.

B5. Simulations for iterative tracing

All simulation programs were written in Mathematica®, for which we reparameterized the model ($\beta_0 = \beta p_c$; $\beta_1 = \beta(1 - p_c)$). For any β_0, τ_{lat}, and δ, 100,000

clusters were simulated and R_c was calculated as the average sum Y of all (partial) infectious periods, multiplied by β_1. Then, β_1 was solved from the equation $R_c = 1 = \beta_1 Y$, and β_0 and β_1 were used to obtain an (R_0^{pre}, p_c*) pair. A series of β_0 values resulted in a series of (R_0^{pre}, p_c*) pairs from which (by interpolation with the SplineFit function in Mathematica®) p_c* could be determined for any R_0^{pre}.

B6. Relations between p_c*, τ_{lat} and δ with iterative tracing

Figure S1 shows the relation between τ_{lat} and p_c* with iterative tracing, in the absence of delays, with approximate positions of the four real infections indicated. The major difference between Figure S1 and Figure 2 (for single-step tracing) is observed for small τ_{lat}, when single-step tracing is often ineffective. Iterative tracing is always effective if all contacts are traced ($p_c = 1$), because in that case the first detection event will instantaneously result in quarantine of all infecteds.

Figure S2 shows the relation between τ_{lat} and δ with iterative tracing for the four special cases, with approximate positions of the four real infections indicated. No p_c* = 1 contour is plotted, because this is impossible to obtain through simulations: the simulated clusters become very large, which takes too much time. Instead, a p_c* = 0.95 contour is drawn.

Comparison of Figures 3 and S2 clearly indicates that iterative tracing is more effective than single-step tracing only if τ_{lat} and δ are such that tracing is on the brink of being effective at all (p_c* ≈ 1). For specific infections this means that iterative tracing allows for an extra tracing delay of one or two days.

Figure S1. The effectiveness of iterative contact tracing without tracing delays. Effectiveness is expressed as the minimum proportion of contacts that need to be traced for effective control (critical tracing probability p_c*). The plots show p_c* as a function of the latent period relative to the mean time to detection (τ_{lat}). There are four special cases: (A) short infectious period and variable time to detection, (B) short infectious period and fixed detection time, (C) long infectious period and variable time to detection, and (D) long infectious period and fixed detection time. The three curves denote p_c* for different values of the pre-isolation reproduction ratio R_0^{pre}. Indicated by dashed lines are the average τ_{lat} for four infections, in the panels with closest correspondence to the actual parameter values (Table 2). Influenza appears in two panels with long and short infectious period, because it corresponds to both parameter sets equally.

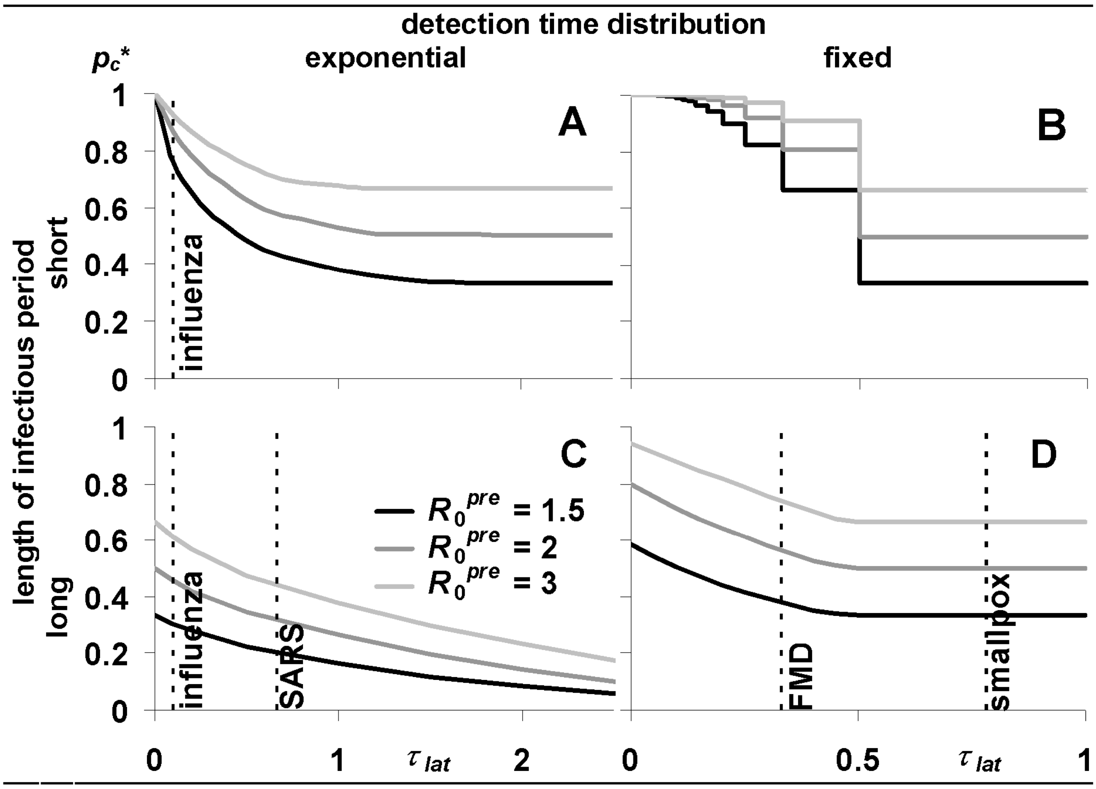

Figure S2. The effectiveness of iterative contact tracing with tracing delays, with the pre-detection reproduction ratio R_0^{pre} = 1.5. Effectiveness is expressed as the minimum proportion of contacts that need to be traced for effective control (critical tracing probability p_c*). The contour plots show p_c* as a function of the tracing delay δ and the latent period τ_{lat}, measured relative to the mean detection time, for four special cases: (A) short infectious period and variable incubation period, (B) short infectious period and fixed incubation period, (C) long infectious period and variable incubation period, and (D) long infectious period and fixed incubation period. Dark grey shadows indicate areas where tracing is almost ineffective (almost, because the black lines do not indicate p_c* = 1), light grey shadows indicate areas where p_c* = 0.33. Indicated by dashed lines are the average τ_{lat} for four infections, in the panels with closest correspondence to the actual parameter values (Table 2).

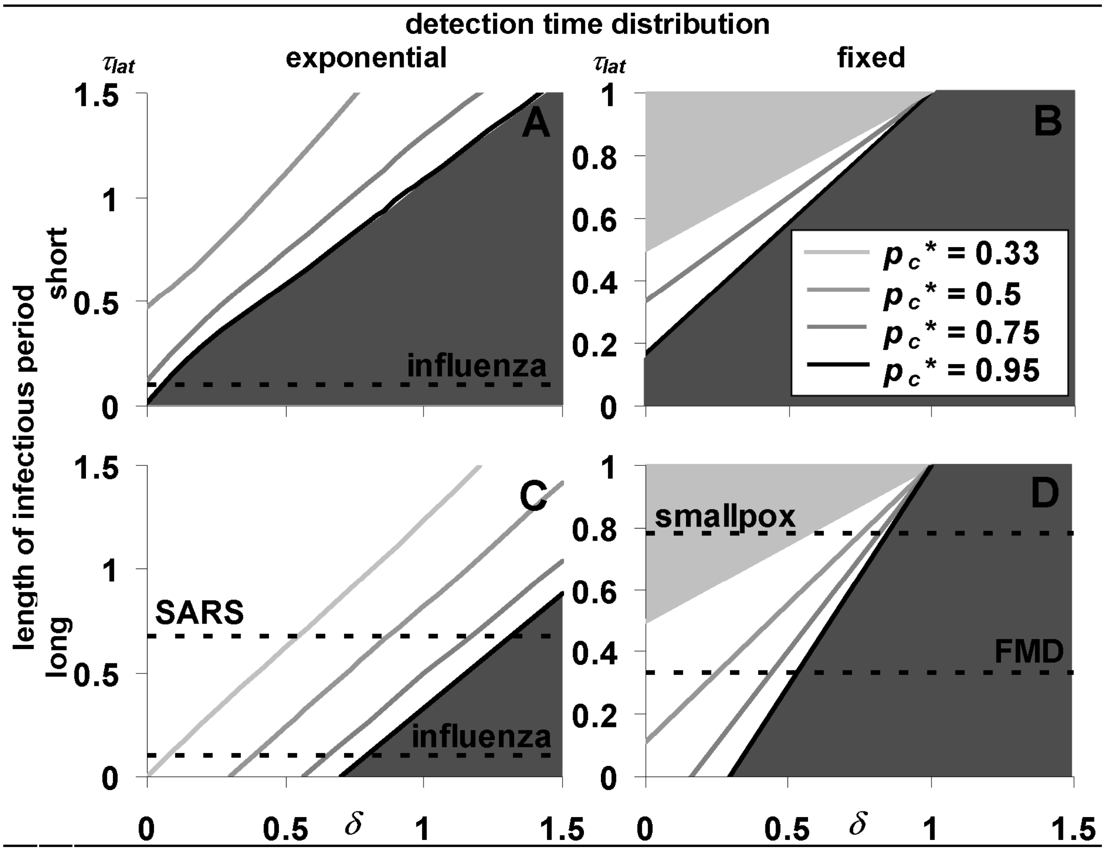

Supplement: Supporting Information — for the paper “The Effectiveness of Contact Tracing in Emerging Epidemics” (0.60 MB PDF) [file pone.0000012.s001.pdf]
